# Dedifferentiated Human Articular Chondrocytes Redifferentiate to a Cartilage-Like Tissue Phenotype in a Poly(ε-Caprolactone)/Self-Assembling Peptide Composite Scaffold

**DOI:** 10.3390/ma9060472

**Published:** 2016-06-17

**Authors:** Lourdes Recha-Sancho, Franklin T. Moutos, Jordi Abellà, Farshid Guilak, Carlos E. Semino

**Affiliations:** 1 Tissue Engineering Laboratory, Bioengineering Department, IQS School of Engineering, Ramon Llull University, Via Augusta 390, Barcelona 08017, Spain; lourdesrechas@iqs.url.edu; 2 Cytex Therapeutics Inc., Durham, NC 27705, USA; frank.moutos@cytextherapeutics.com (F.T.M.); guilak@wustl.edu (F.G.); 3 Department of Orthopaedic Surgery, Washington University and Shriners Hospitals for Children—St. Louis, St. Louis, MO 63110, USA; 4 Analytical Chemistry Department, Institut Químic de Sarrià, Ramon Llull University, Via Augusta 390, Barcelona 08017, Spain; jordi.abella@iqs.url.edu

**Keywords:** cartilage tissue engineering, 3D cell culture, human chondrocytes, cell differentiation, biomimetic materials

## Abstract

Adult articular cartilage has a limited capacity for growth and regeneration and, with injury, new cellular or biomaterial-based therapeutic platforms are required to promote repair. Tissue engineering aims to produce cartilage-like tissues that recreate the complex mechanical and biological properties found *in vivo*. In this study, a unique composite scaffold was developed by infiltrating a three-dimensional (3D) woven microfiber poly (ε-caprolactone) (PCL) scaffold with the RAD16-I self-assembling nanofibers to obtain multi-scale functional and biomimetic tissue-engineered constructs. The scaffold was seeded with expanded dedifferentiated human articular chondrocytes and cultured for four weeks in control and chondrogenic growth conditions. The composite constructs were compared to control constructs obtained by culturing cells with 3D woven PCL scaffolds or RAD16-I independently. High viability and homogeneous cell distribution were observed in all three scaffolds used during the term of the culture. Moreover, gene and protein expression profiles revealed that chondrogenic markers were favored in the presence of RAD16-I peptide (PCL/RAD composite or alone) under chondrogenic induction conditions. Further, constructs displayed positive staining for toluidine blue, indicating the presence of synthesized proteoglycans. Finally, mechanical testing showed that constructs containing the PCL scaffold maintained the initial shape and viscoelastic behavior throughout the culture period, while constructs with RAD16-I scaffold alone contracted during culture time into a stiffer and compacted structure. Altogether, these results suggest that this new composite scaffold provides important mechanical requirements for a cartilage replacement, while providing a biomimetic microenvironment to re-establish the chondrogenic phenotype of human expanded articular chondrocytes.

## 1. Introduction

Articular cartilage is an avascular tissue with a highly specialized extracellular matrix (ECM) architecture and composition that allows it to withstand the mechanical requirements of the diarthrodial joint [1]. The principal function of cartilage is to withstand mechanical loads while allowing low friction movements of joints over millions of cycles of loading [2]. Chondrocytes are the only resident cells in articular cartilage and, therefore, are responsible for synthesizing and maintaining the complex ECM [3]. However, cartilage shows little or no capacity for self-repair, and cartilage defects generated by trauma or injury can result in long-term pain and loss of joint function. Moreover, due to lack of a healing response, such focal injuries can often lead to progressive degenerative changes that compromise joint function [4]. While several surgical methods are currently used to enhance cartilage repair [5], these procedures have not shown long-term clinical success. Therefore, new strategies for cartilage repair are required and tissue engineering has emerged as a potential source to generate cartilage-like structures through the use of cells and biomaterials [6,7].

One approach of cartilage tissue engineering (CTE) is based on mimicking the natural tissue environment in order to stimulate the formation of new cartilage [8,9]. In this case, biomimetic materials which are similar structurally and mechanically to ECM and recreate *in vivo* conditions are of high interest [10,11]. A wide variety of scaffolds have been explored so far in CTE and they can be classified into natural or synthetic biomaterials [12]. Natural materials include collagen, fibrin, alginate or hyaluronan among others, and they possess a variety of properties, such as biocompatibility and biodegradability. However, they present variability from batch to batch and possible modifications to improve them are limited. In contrast, the main advantages of synthetic biomaterials are their reproducibility and their design with specific mechanical, structural, and biological properties. Some examples include polyethylene glycol (PEG), polyurethanes, polycaprolactone (PCL), and self-assembling peptides [6]. Additionally, composites scaffolds consisting of two or more biomaterials with different properties have been studied to provide enhanced properties that cannot be achieved with a single material [13]. In the present work, a new composite was generated by combining two different synthetic biomaterials: the three-dimensional (3D) woven poly (ε-caprolactone) (PCL) scaffold and the RAD16-I self-assembling peptide. 3D weaving can be used to create porous structures arranged in multiple layers of continuous fibers in three orthogonal directions, as previously described [14]. Such scaffolds were engineered with predetermined properties aiming to reproduce the anisotropy, viscoelasticity, and tension–compression non-linearity of native articular cartilage. Moreover, PCL is a Food and Drug Administration (FDA) approved biomaterial, biocompatible and biodegradable with extended experience medical use [15,16]. RAD16-I self-assembling peptide is commercially available under the name of PuraMatrix™ [17]. It is a water soluble peptide that self-assembles into a network of nanofibers when the ionic strength increases or when the pH is adjusted to neutrality forming a soft hydrogel [18]. The cells can be embedded in a truly 3D matrix during the self-assembling process driven by weak non-covalent interactions including hydrogen bonds, ionic bonds, electrostatic interactions, van der Waals interactions, *etc.* [19]. Importantly, these weak interactions allow cells to freely migrate, interact and extend different cellular processes. Therefore, this nanofiber network promotes cell–cell and cell–matrix interactions allowing cells to freely grow, proliferate and differentiate under specific experimental conditions [18,20]. Previous studies showed the ability of RAD16-I to support cell maintenance in a variety of cellular types, including endothelial cells, hepatocytes, neural cells, fibroblasts, osteoblasts, embryonic, and somatic stem cells [21,22,23,24,25,26,27]. It is a biocompatible and biodegradable peptide that can be defined as “non-instructive” from the point of view of cell receptor recognition/activation, since it does not contain specific motifs in their native sequence [20]. Moreover, it can be functionalized with specific signaling motifs (or molecules) to promote different cellular responses [28,29,30].

The strategy in this study was to combine these two dissimilar materials in order to facilitate the attachment, proliferation, and differentiation of embedded cells, while simultaneously providing a biomimetic mechanical environment of the native tissue [31]. Previous studies have shown the use of both biomaterials in CTE applications. In previous studies, PCL scaffolds were combined with other hydrogels, such as agarose, fibrin, Matrigel^®^, or interpenetrating network (IPN) gels to mimic the functional properties of cartilage while providing a cellular environment that is conducive to chondrogenesis for different cellular types [14,32,33,34]. As each type of hydrogel may possess significantly different biological and biomechanical properties (e.g., matrigel *vs.* IPN “toughgels”), the 3D composite of a woven fiber scaffold infiltrated with a gel matrix can be tailored to provide different properties at the macroscopic as well as the cellular scale. RAD16-I, for example, has been shown to induce spontaneous chondrogenic commitment of mouse embryonic fibroblasts that were cultured in the peptide scaffold [35,36]. Moreover, RAD16-I can also promote cartilage differentiation of human dermal fibroblasts under chemical induction [37]. Although the stiffness of the scaffold can be modulated by changing the peptide concentration, this hydrogel provides a soft and permissive environment where cells can act remodeling the surrounding matrix. Therefore, cells are embedded in a dynamic microenvironment and the mechanical properties of the entire construct can evolve during the culture time, ending in a stiffer structure [27].

Besides the scaffold, determining the optimal cell source for CTE is still a challenge. Adult human chondrocytes represent a potential cell source, because they are found in native cartilage and have the original chondrogenic phenotype. Since they are isolated from patients, the main drawback is to obtain a sufficient cell number for their use in clinics. For this reason, an *ex vivo* expansion often is required to overcome the limited supply [38], but chondrocytes dedifferentiate in monolayer cultures losing their ability to express articular cartilage ECM specific markers [39,40]. Therefore, the recovery of the chondrogenic phenotype is an essential step prior to further application. 3D matrices providing environmental support and mimicking the native tissue architecture have emerged as a potential toolbox in CTE platforms [41]. Several studies showed the use of 3D cultures to enable the *in vitro* maintenance of chondrocyte phenotype [42,43,44,45]. The goal of the present study is to stimulate the redifferentiation of human expanded chondrocytes into functional cartilage-like tissue using a new PCL/RAD composite. Their dedifferentiation during monolayer expansion has been previously demonstrated [46]. We hypothesize that the biochemical and biomechanical signals provided by the composite scaffold could guide chondrocytes commitment to re-establish and maintain its mature cartilage phenotype.

## 2. Results

### 2.1. PCL/RAD Composite Development

**Figure 1:**
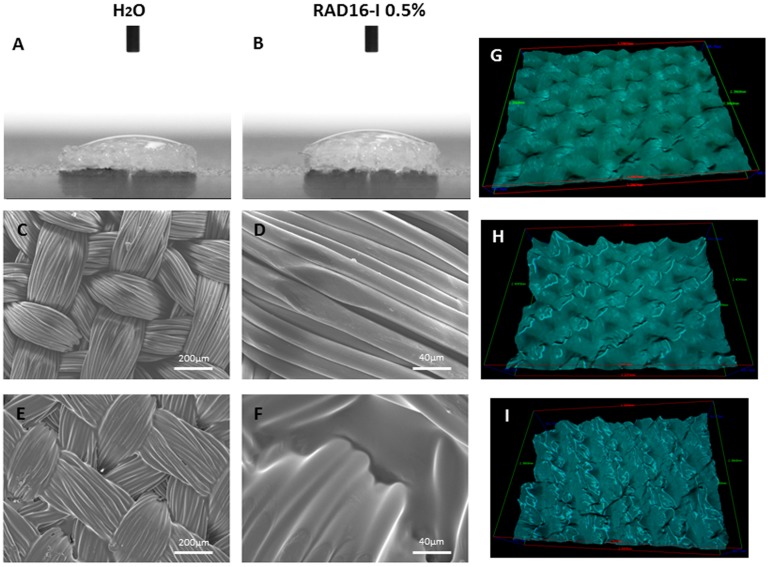
Wettability and fiber architecture of woven poly (ε-caprolactone) (PCL) scaffold and PCL/RAD16-I self-assembling peptide composite scaffold. (**A**) Water contact angle; (**B**) RAD16-I 0.5% solution contact angle; (**C**) Surface view of PCL structure by scanning electron microscope (SEM); (**D**) Close up of panel C of microfiber detail; (**E**) Surface view of composite PCL/RAD by SEM. RAD16-I 0.5% was lyophilized within the PCL scaffold; (**F**) Close up of panel E; (**G**) Three-dimensional (3D) view of PCL scaffold surface; (**H**) 3D view of PCL scaffold surface wet with water; (**I**) 3D view of PCL/RAD composite scaffold surface.

As a previous step a simple wettability assay was performed on PCL scaffold measuring the contact angle formed with water (Figure 1A) and RAD16-I at 0.5% (Figure 1B). In both cases, the liquid drop was totally absorbed by the PCL scaffold (contact angle << 90°) indicating high wettability. Then, the fiber architecture of the woven PCL scaffolds and PCL/RAD composite scaffold were assessed by scanning electron microscope (SEM) (Figure 1C–F). In the case of the composite, areas of RAD16-I peptide deposition could be observed within the highly organized woven morphology of the fiber scaffold (Figure 1E,F). Moreover, the grooves observed in the woven PCL scaffold surface can be easily filled with water and RAD16-I peptide solution, as evidenced by the 3D view on stereoscopic microscope (Figure 1G–I). Altogether, our results show thorough infiltration of the peptide solution into the 3D structure of PCL scaffold.

### 2.2. Chondrocyte Viability during Culture in 3D-Scaffolds

Next, we evaluated the seeding capacity of Articular Chondrocytes (AC) into three different scaffolds: PCL, RAD16-I and its combination (composite PCL/RAD16-I). The composite scaffold was studied in parallel with the simple scaffolds (PCL and RAD16-I) to compare the different platforms and to ascertain if the mixture implies an improvement from the point of view of cellular behavior and chondrogenic differentiation.

**Figure 2:**
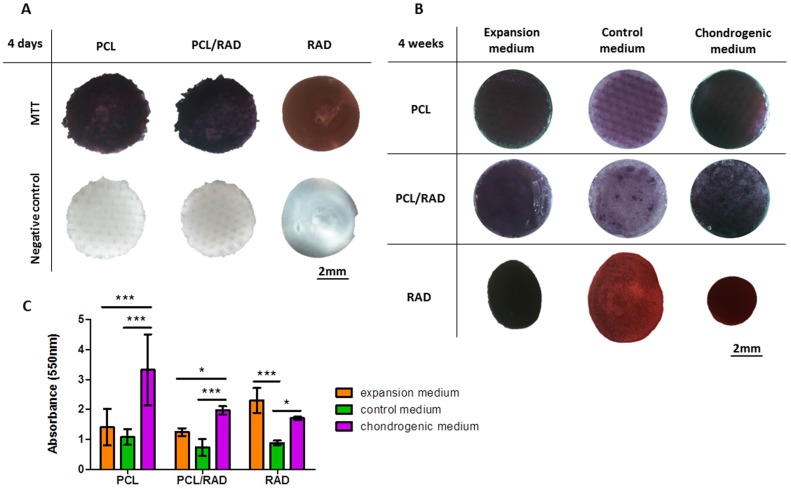
Viability of Articular Chondrocytes (AC) cultured in 3D scaffolds at different time points. PCL, PCL/RAD composite, and RAD scaffolds were seeded with AC and incubated with MTT to assess cell viability and distribution. (**A**) Construct appearance after MTT incubation at 4 days of culture with expansion medium. The same constructs were incubated in the absence of MTT reagent as a negative control; (**B**) Construct appearance after MTT incubation at 4 weeks of culture with the different culture media: expansion, control and chondrogenic media; (**C**) MTT values at 4 weeks of culture were expressed of formazan (product) absorbance at 550 nm. (Statistical differences are indicated as: * for *p* < 0.05, ** for *p* < 0.01, and *** for *p* < 0.001, Two-way ANOVA, *N* = 2 *n* = 3).

AC were expanded and dedifferentiated in monolayer, as published in a previous work [46]. Then, cells were seeded into each scaffold system to obtain the corresponding constructs. First, MTT (3-[4,5-dimethylthiazol-2-yl]-2,5-diphenyl tetrazolium bromide) was performed to assess cellular viability in each construct at two different time points: 4 days and 4 weeks (Figure 2). At 4 days of culture in expansion media, cells remained alive inside the constructs. Moreover, AC were equally distributed as observed by the homogeneity of the purple color among the constructs (Figure 2A). However, the experiment at 4 weeks was more complex since two more culture media (control and chondrogenic) were added to perform further chondrogenic evaluation assays. Then, each construct type was cultured in three different culture media: expansion, control, and chondrogenic (see Materials and Methods). Viability results after 4 weeks of culture indicated a good cell survival for all construct and media tested (Figure 2B). Nevertheless, higher values of absorbance were observed for PCL and PCL/RAD constructs cultured with chondrogenic medium compared to expansion and control media (Figure 2C). In contrast, for RAD construct no differences were observed between expansion and chondrogenic media but control medium presented the lowest values (Figure 2C). As observed by color distribution at 4 weeks of culture, the distribution of cells in the construct was homogeneous for all cases (Figure 2B).This event was also before observed at 4 days of culture (Figure 2A). Moreover, the intensity of the purple color was higher in the case of expansion and chondrogenic media compared to control medium, which correlates with the absorbance values.

### 2.3. Chondrogenic Differentiation of Chondrocytes Seeded in 3D-Scaffolds

Further analysis was focused on assessing the potential of the three construct types to support chondrogenic differentiation with AC. It is important to note the basic composition differences among the media used, since we aimed to chemically induce the chondrogenic differentiation process by using chondrogenic medium containing TGF-β1, L-ascorbic acid 2-phosphate, and dexamethasone as inducers [47]. Additionally, the expansion medium containing FBS and different growth factors used to culture AC in monolayer was also used in the culture of the 3D constructs.

**Figure 3:**
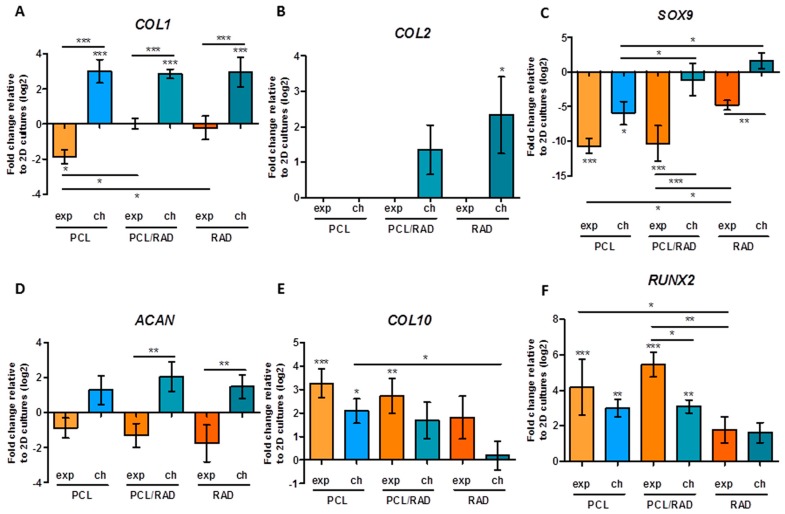
Gene expression levels of chondrogenic and hypertrophic markers of Articular Chondrocytes (AC) cultured in 3D scaffolds over 4 weeks. AC in PCL, PCL/RAD and RAD scaffolds were analyzed for gene expression in expansion (exp) and chondrogenic (ch) media. Collagen type I (COL1, **A**); collagen type II (COL2, **B**); SOX9 (**C**); aggrecan (ACAN, **D**); collagen type X (COL10, **E**); and RUNX2 (**F**) were determined through qRT-PCR. Ct values relative to ribosomal protein L22 (RPL22) were obtained and reported as fold increase (ΔΔCt) relative to 2D cultures in expansion medium (baseline) (Statistical differences are indicated as: * for *p* < 0.05, ** for *p* < 0.01, and *** for *p* < 0.001, One-way ANOVA, *N* = 2 *n* = 3).

Gene expression pattern studies were performed from the point of view of protein and gene expression. Regarding gene expression profiles, COL1, COL2, COL10, ACAN, SOX9, RUNX2, and RPL22 as housekeeping gene were analyzed by quantitative Reverse Transcription Polymerase Chain Reaction (qRT-PCR) at 4 weeks of culture (Figure 3). In the case of constructs cultured with control medium, RNA levels were not enough to perform the qRT-PCR. COL1 expression, as a marker of dedifferentiation, was reduced in PCL constructs cultured with expansion medium, maintained equal to 2D cultures levels (used to normalize expressions levels, baseline) in PCL/RAD and RAD scaffolds cultured with expansion medium and increased in all scaffolds cultured with chondrogenic medium (Figure 3A). Interestingly, the expression of COL2, one of the main components of articular cartilage, was only slightly increased in PCL/RAD and RAD scaffolds cultured with chondrogenic medium; however, only for RAD scaffold was significance difference detected (Figure 3B). The expression of the transcription factor SOX9, as a marker of chondrogenesis, was down-regulated in PCL cultured with both media and in PCL/RAD constructs cultured with expansion medium. However, it was maintained similar to 2D baseline levels in PCL/RAD constructs cultured with chondrogenic medium and in RAD constructs (Figure 3C). In the case of aggrecan (ACAN), its expression was reduced in all constructs cultured with expansion medium and increased in all constructs cultured with chondrogenic medium, but no significant differences were detected relative to baseline. Nevertheless, differences could be observed between expansion and chondrogenic medium in PCL/RAD scaffolds and in RAD constructs (Figure 3D). Regarding hypertrophic markers, COL10 and RUNX2 expression were up-regulated in some of the constructs with respect to baseline, but, importantly, no significant increase was detected in RAD constructs and in PCL/RAD constructs cultured with chondrogenic media in the case of COL10 expression (Figure 3E,F).

**Figure 4:**
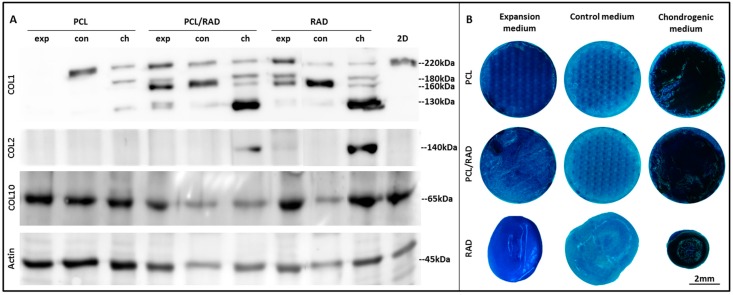
Protein expression characterization of Articular Chondrocytes (AC) cultured in the 3D scaffolds after 4 weeks of culture. (**A**) Western blot results of collagen type I, II and X when AC were maintained in expansion (exp), control (con) and chondrogenic (ch) media in the different scaffolds (PCL, PCL/RAD, and RAD) and in monolayer. Actin expression was used as an internal control. Samples were prepared in triplicate; (**B**) Toluidine blue staining of AC seeded into PCL, PCL/RAD, and RAD scaffolds cultured in expansion, control and chondrogenic media.

Moreover, collagens type I, II, and X were analyzed by Western blot in monolayer (2D) and in the different 3D systems (Figure 4A). Collagen type I (COL1) was observed in the 2D cultures and in all 3D constructs; except for the case of PCL constructs cultured with expansion medium, correlating with the down-regulation detected by qRT-PCR (see Figure 3A). Interestingly, the bands pattern was different between samples producing COL1. A band of high molecular weight (~220 kDa), probably a pro-collagen intermediate, was detected in 2D and in 3D cultures positive samples. In addition, more bands of lower molecular weight (ranging from 180 to 130 kDa) were observed in 3D cultures of PCL/RAD and RAD (in all media composition) and PCL in chondrogenic conditions. Remarkably, collagen type II (COL2) was only detected in PCL/RAD and RAD construct cultured with chondrogenic medium, correlating with gene expression results (see Figure 3B). Collagen type X (COL10) protein expression was detected in all samples, both 2D cultures and 3D constructs, which correlates with the expression patterns obtained by qRT-PCR (see Figure 3E).

Furthermore, AC constructs were stained with toluidine blue to asses qualitatively the production of glycosaminoglycans (GAGs) by the cells (Figure 4B). Constructs cultured with chondrogenic medium became highly blue stained, indicating a significant production and accumulation of GAGs. Constructs cultured with expansion media showed less staining for GAGs than previous ones and finally, those cultured in control media were relatively weak stained.

### 2.4. SEM Characterization of AC Constructs

**Figure 5:**
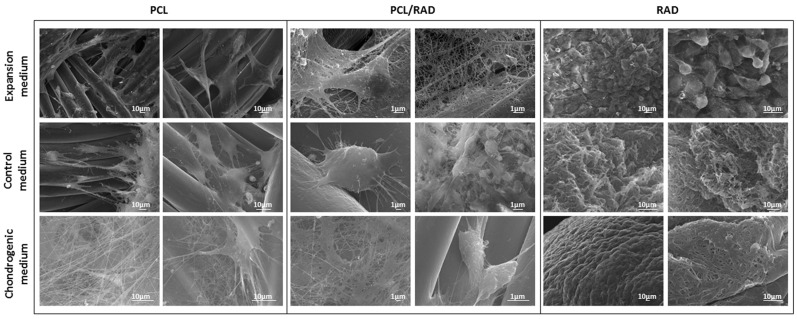
Scanning Electron Microscopy (SEM) images of Articular Chondrocytes (AC) cultured in 3D scaffolds after 4 weeks. AC were seeded into PCL, PCL/RAD, and RAD scaffolds and cultured with expansion, control, and chondrogenic media. Two images per condition were shown.

At microscopic level, SEM images from the different culture conditions of AC were obtained in order to evaluate the morphology of the cells and their interaction with the different scaffolds (Figure 5). AC in PCL scaffolds appeared elongated, growing onto the surface of PCL fibers and, interestingly, more fibers were observed under chondrogenic conditions probably due to an increase in the ECM components secretion. Regarding PCL/RAD constructs, cells appeared to be well attached to the PCL fibers, with a more spherical shape than PCL alone. Finally, AC cultured in RAD scaffolds presented in general a spherical morphology and more cellular density due to the construct condensation occurred during the culture (see Figure 2B).

### 2.5. Mechanical Testing

**Figure 6:**
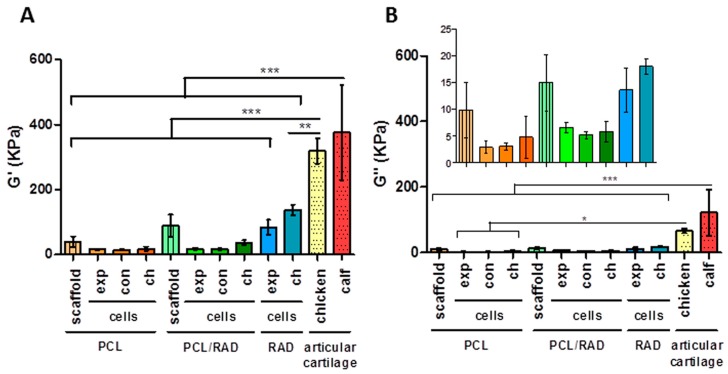

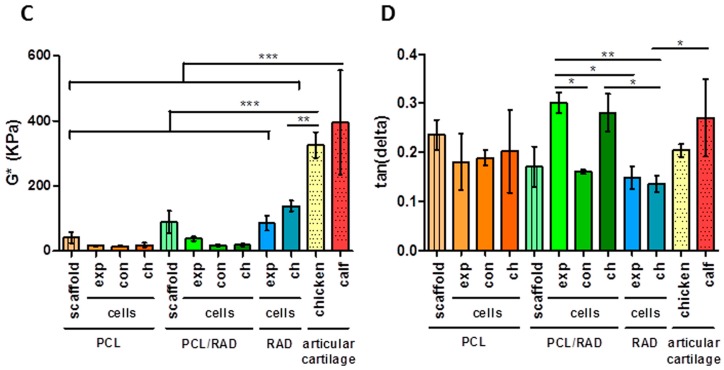
Mechanical characterization of PCL-based scaffolds and Articular Chondrocytes (AC) 3D constructs after 4 weeks of culture. (**A**) Storage modulus (G’) measures the sample’s elastic behavior; (**B**) Loss modulus (G’’) measures the viscous response of the material; (**C**) Complex modulus (G*) is the sum of storage and loss modulus; (**D**) Tan (delta) is the ratio of the loss to the storage. PCL scaffold and PCL/RAD composite scaffold refers to the acellular scaffold. 3D AC constructs cultured in expansion (exp), control (con), and chondrogenic (ch) media. Small pieces of articular cartilage of chicken and calf were measured in the same conditions (Statistical differences are indicated as: * for *p* < 0.05, ** for *p* < 0.01, and *** for *p* < 0.001, One-way ANOVA, *N* = 2 *n* = 3).

Viscoelastic properties of the scaffolds alone and their AC seeded constructs were assessed by dynamic mechanical analysis (DMA) at 4 weeks of culture and compared to natural articular cartilage (Figure 6). Regarding the elastic component (G’, storage modulus), native articular cartilage from chicken and calf displayed significantly higher storage values than the 3D scaffolds and constructs studied (Figure 6A). No significant differences were observed in storage modulus between scaffolds and constructs groups. The viscous component (G’’, loss modulus) presented a similar tendency to the storage modulus: calf native cartilage differed from all the studied 3D scaffolds and chicken articular cartilage only presented differences with cellular PCL scaffolds (Figure 6B). All samples presented G’ values much higher than G’’ values meaning that the measured material was more elastic than viscous. Therefore, as the complex modulus (G*) is the sum of both components, in this case, G* basically corresponds to the elastic component and presented the same pattern as the storage modulus (Figure 6C). Finally, a different tendency was observed in tan delta, which gives us an idea of the full mechanical response of the material (Figure 6D). The studied scaffold and constructs were closely related to native articular cartilage; except in the case of RAD chondrogenic constructs with calf articular cartilage where differences exist. Moreover, statistical differences could be observed between PCL/RAD and RAD constructs when the same medium was used. Therefore, the combination of PCL scaffolds and RAD16-I hydrogel changed their viscoelastic nature after 4 weeks of culture with AC, as reflected in the tan delta values of the composite which were increased compared to RAD scaffolds alone. This effect was not present between the composites PCL/RAD and the PCL scaffolds groups. Moreover, there were no differences between cellularized and acellular PCL scaffolds, but surprisingly, there were significant differences between PCL/RAD scaffold and PCL/RAD with cells in expansion and chondrogenic media. Moreover, cellularized PCL/RAD scaffolds reached equivalent tan (delta) values to native articular cartilage. This fact might indicate that the combination of scaffolds and cells provide the optimal working conditions in this particular configuration.

## 3. Discussion

In the present work, two well-established scaffolds were combined to create a new composite biomaterial with novel biomechanical and biological properties for CTE applications. A 3D woven PCL scaffold and the RAD16-I self-assembling peptide nanofiber hydrogel were selected based on their reported properties in extended 3D culture studies [14,18]. This approach provides biomimetic mechanical properties from the PCL scaffold and biological/chemical stimulation from the hydrogel RAD16-I in the composite matrix. In particular, the developed PCL/RAD scaffold was used to assess the capacity of the composite to re-establish chondrogenic phenotype of expanded dedifferentiated human articular chondrocytes (AC). In earlier studies, both biomaterials were used independently as scaffolds for *in vitro* chondrocyte culture studies [48,49]. Moreover, the woven PCL scaffold was previously infiltrated with other biomaterials, such as fibrin or Matrigel, to enhance its functionality [13,34,50]. However, it is important to note that different gel matrices may have different biological or biomechanical properties, particularly in combination with a 3D fiber structure. For example, Matrigel has been shown to be conducive to chondrogenic induction of MSCs, but may contain components that induce an immunogenic response [51]. Conversely, IPN hydrogels possess extremely tough mechanical properties and their composition is well-defined, but may influence cell proliferation and metabolic activity [52]. Therefore, using this general approach, a new composite scaffold incorporating both micro- and nano-scale features was developed and evaluated to foster chondrogenesis.

Due to high wettability properties of the PCL scaffold (Figure 1A,B), RAD16-I peptide combined with cells was easily introduced between the interweaving fibers of the scaffold. A comparative study was performed in order to contrast the properties of each scaffold alone (3D woven PCL scaffold or RAD16-I self-assembling peptide) and the corresponding composite (PCL/RAD). Moreover, three culture media compositions were evaluated (expansion, control and chondrogenic) to study the response of AC to different conditions. In general, good performance was observed in all the conditions studied, but some important differences were detected. Viability results showed the lowest values in constructs cultured in control medium (Figure 2), which is expected due to the lack of growth factors or serum in the medium (see *2.5. 3D Cell Culture Techniques*). In terms of gene expression profile, significant differences were observed between expansion and chondrogenic media (Figure 3). As expected, the chondrogenic factors added to the medium induced an apparently chondrogenic phenotype with combined expressions of the different collagens studied [53]. Regarding the scaffold system, the presence of RAD16-I peptide (in the composite or alone) enhanced the expression of cartilage markers as evidenced by up-regulation of COL2 and ACAN and maintenance of SOX9 compared to 2D culture levels. However, COL1 and COL10 were also upregulated which could indicate a partial redifferentiation and a possible mechanism of presumptive hypertrophy. In terms of protein expression, COL1 presented different band patterns between samples, suggesting a possible protein maturation process [54]. Considering the expected size for mature COL1 (139 kDa for α1 chain and 129 kDa for α2 chain), the scaffolds containing RAD16-I (composite or alone) under chondrogenic induction expressed higher quantity of the mature COL1 (130–140 kDa) compared to other conditions tested (Figure 4A). This result indicates that COL1 protein was processed differentially by influence of the medium composition and the scaffold system, and only under specific conditions was the final mature product obtained contributing to the formation of a more physiological matrix composition/structure. It should be emphasized that COL2, characteristic of chondrogenic differentiation, was only detected in PCL/RAD and RAD scaffolds cultured with chondrogenic medium, which suggest that the expression of this characteristic cartilage protein expression was due to the presence of RAD16-I hydrogel in the matrix. Curiously, these two construct systems also present the most severe degraded profile of COL1, which could indicate the presence of some mechanism to increase the ratio of COL2/COL1, a characteristic of cartilage tissue. Toluidine blue staining evidenced the synthesis and accumulation of some considerable amounts of proteoglycans, presumably aggrecan (or others unidentified), favored under chondrogenic conditions (Figure 4B). Expansion and control media did not promote such level of staining, which suggest that dedifferentiated AC do not re-express the differentiated phenotype without chondrogenic inducers in the medium [29].

Further, the mechanical testing revealed that PCL-based constructs (PCL and PCL/RAD with cells) after 4 weeks of culture retained the same viscoelasticity displayed by PCL scaffolds alone (Figure 6). In contrast, initial values of the RAD scaffold alone could not be measured under the same conditions due to the soft nature of the peptide. However, previous studies described that the concentration at which AC were seeded initially (0.15% (w/v) RAD16-I) corresponds to 100 Pa [55]. This indicates that the mechanical properties of the constructs were evolving during culture time, ending with a stiffer and compacted structure (~100 kPa). The RAD scaffold provides a soft and permissive microenvironment where cells can extend different cellular processes as they elongate and form networks, leading to the spontaneous contraction of the 3D construct and changing the initial viscoelastic properties. Finally, due to the disparity in mechanical properties presented among constructs types at 4 weeks of culture (PCL, PCL/RAD, and RAD), the measurement condition settings were adjusted to obtain comparable data from different scaffold platforms (see *2.10. Mechanical Characterization*). Interestingly, at the end of the culture, all the constructs tested seem to present a behavior compatible to systems undergoing chondrogenesis, since the viscoelastic nature (tan delta = G’’/G’) was similar to native articular cartilage. However, significant differences exist in the elastic component (G’ values), since the obtained values in the 3D cultures differ several folds from measured native cartilage. In fact, the surrounding microenvironment determines cell growth and differentiation, since cells have the ability to actively sense and react to the properties of the extracellular matrix [56,57]. Therefore, mechanical properties of 3D scaffolds have significant influence on regulating cell activities and controlling these properties has a key role in future applications of engineered constructs [58,59].

These results propose a novel 3D composite scaffold, which is designed to mimic the cartilage matrix, to promote redifferentiation of dedifferentiated human chondrocytes under induction conditions. The nanoscale microenvironment provided by RAD16-I scaffold and the microscale mechanical properties provided by fibers of PCL represent a realistic approach, aiming to recreate a transient complex 3D architecture found in native cartilage. Previous studies have reported the successful combination of PCL with other materials such as fibrin, alginate, and poly-acrylamide [32,33,34]. Here, we hypothesized that the synergistic properties obtained by the combination of the woven PCL scaffolds with the self-assembling peptides for CTE would significantly improve the biological as well as biomechanical properties of engineered cartilage constructs. Moreover, the developed composite could have advantages when used in the context of defect implant in an acute injury scenario, basically due to the biomimetic initial mechanical properties provided by the reinforcing PCL component.

## 4. Materials and Methods

### 4.1. Scaffold Production

Scaffolds were woven from multifilament PCL yarns (EMS-Griltech, Domat, Switzerland) as previously described [33]. For this study, 11 layers of yarns were axially oriented in alternating x and y directions with a third set of fibers passing through the thickness of the structure (z-direction). The result is a 0.75 mm thick structure with a total internal void space of ~45% comprised of interconnected rectangular pores measuring approximately 0.35 mm × 0.25 mm × 0.1 mm. Scaffolds were treated with 4 M NaOH (Sigma-Aldrich, St. Louis, MO, USA) for 16 h to clean the fibers and increase surface hydrophilicity, rinsed in deionized H_2_O, and dried. Scaffolds were subsequently heat set for 10 min at 57 °C in deionized H_2_O. Dried scaffolds were then punched using a trephine to obtain uniform 5 mm disks. For tissue engineering experiments, disks were ethylene-oxide sterilized in 24 well ultra-low attachment plates (Corning, Corning, NY, USA) prior to use.

### 4.2. Contact Angle Measurements

The contact angles of water and RAD16-I over the surface of PCL scaffold were measured in a goniometer (DSA 100, Kruss, Germany). 2 µL drop of Milli-Q water or RAD16-I 0.5% (w/v) (PuraMatrix™, 354250, Corning, NY, USA) was placed over the surface of the scaffold and a camera immediately acquired an image. Measurements were made at room temperature and three repetitions per each condition were performed.

### 4.3. Scanning Electron Microscopy (SEM)

The topographies of PCL scaffolds and PCL/RAD composite scaffolds were examined under field emission SEM (JOEL JSM-5310, JOEL, Peabody, MA, USA) at an accelerating voltage of 20 kV. Previously, the surface of the scaffolds were gold-coated (thickness ~150 Å) using a Polaron SC7620 Sputter Coater (Quorum Technologies, East Sussex, UK).

Briefly, constructs after 4 weeks of culture were fixed in 2% (w/v) paraformaldehyde (P6148 Sigma-Aldrich, St. Louis, MO, USA) and dehydrated in successive ethanol (51976, Sigma-Aldrich, St. Louis, MO, USA) washes. Once dehydrated, samples were dried using a CO_2_ critical point dryer (Emitech K850, Quorum Technologies, East Sussex, UK). Then, dried samples were subsequently coated with a thin layer of graphite (Emitech K950X, Quorum Technologies, East Sussex, UK). Finally, samples were examined under JEDL J-7100 field emission scanning electron microscope (JOEL, Peabody, MA, USA) (Cathodeluminiscence spectrometer GATAN MONO-CL4, EDS detector, retroscattered electron detector) at an accelerating voltage of 15 kV and 20 kV.

### 4.4. Scaffold Surface Morphology Evaluation

The wettability and fiber architecture of PCL scaffold were evaluated with a stereoscopic microscope (Leica M165 C, Barcelona, Spain). A 20 µL drop of Milli-Q water or RAD16-I 0.5% (w/v) was placed over the surface of the scaffold and then, they were inspected in a stereoscopic microscope which allows 3D view.

### 4.5. 2D Culture of Human Articular Chondrocytes (AC)

AC cells (CC-2550, Lonza, Basel, Switzerland) were cultured at the recommended seeding density (10,000 cells/cm^2^) from passage 2 to passage 6 in 25 cm^2^, 75 cm^2^, and 175 cm^2^ culture flasks. The expansion medium was composed by Chondrocyte Basal Medium (CC-3217, Lonza, Basel, Switzerland) plus SingleQuots of Growth Supplements (CC-4409, Lonza, Basel, Switzerland) that contain R3-IGF-1, bFGF, transferrin, insulin, FBS and gentamicin/ amphotericin-B. Cultures were maintained in the incubator in humidified atmosphere at 37 °C and 5% CO_2_.

### 4.6. 3D Cell Cultures Techniques

AC at passage 6 were seeded into three different scaffold systems: woven PCL scaffold, RAD16-I self-assembling peptide and its combination (composite PCL/RAD16-I).

In the case of PCL scaffold alone, the procedure consisted of seeding a cell suspension of 25 million cells/mL of AC in expansion medium onto the surface of 5 mm × 0.75 mm woven PCL scaffolds (500,000 cells/scaffold). After 2 h, 100 µL of expansion or control medium were slowly added into the well and, after 4 h, 700 µL were finally added. Control medium was prepared with DMEM, High Glucose, GlutaMAX (61965, Gibco, Thermo Fisher Scientific, Waltham, MA, USA), ITS + Premix 100× (354352, BD Bioscience, San Jose, CA, USA), 100 U/mL Penicillin/100 μg/mL Streptomycin (P11-010, PAA), 40 μg/mL L-Proline (P5607, Sigma-Aldrich, St. Louis, MO, USA) and 1 mM Sodium Pyruvate (11360, Life Technologies, Thermo Fisher Scientific, Waltham, MA, USA).

To obtain AC 3D constructs with the peptide scaffold, a cell suspension of 4 million cells/mL AC in 10% (w/v) sucrose (S0389, Sigma-Aldrich, St. Louis, MO, USA) was mixed with an equal volume of 0.3% RAD16-I (PuraMatrix™, 354250, Corning, NY, USA). Then, the cell-peptide suspension was loaded into wells of 48-culture plate previously equilibrated with 150 µL of control or expansion medium (160,000 cells/encapsulation). The medium induced the self-assembly of RAD16-I and cells were embedded and homogenously distributed into the scaffold. After 30 min, 650 µL of fresh medium was added into the well.

The composites PCL/RAD were performed by mixing a cell suspension of 50 million cells/mL of AC in 10% (w/v) sucrose with 1% (w/v) RAD16-I peptide (1:1) and the homogeneous mixture was seeded onto the woven PCL scaffold disks (500,000 cells/scaffold). Then, 40 µL of expansion or control medium was added and the gel was spontaneously formed inside the PCL scaffolds and the cells were embedded. After 30 min, 60 µL of medium was added in the well and, after 2 h, 700 µL was finally added.

3D cell cultures were maintained in the incubator at 37 °C and 5% CO_2_ over 4 weeks. The medium was changed every second day by removing 400 µL of medium from the well and adding 400 µL of fresh medium. Cultures for chondrogenic differentiation were induced at day 2 with chondrogenic medium (control medium with 10 ng/mL recombinant human transforming growth factor-β1 (TGFβ1) (GF111, Millipore, Billerica, MA, USA), 25 μg/mL L-Ascorbic Acid 2-phosphate (AA2P) (A8960, Sigma-Aldrich, St. Louis, MO, USA) and 100 nM Dexamethasone (D8893, Sigma-Aldrich, St. Louis, MO, USA). After 4 weeks of culture, 3D constructs were analyzed for morphology, viability, gene and protein expression, staining and mechanical characterization.

### 4.7. MTT Assay

MTT [3-(4,5-dimethylthiazol-2-yl)-2,5-diphenyltetrazolium bromide] (M5655, Sigma-Aldrich, St. Louis, MO, USA) assay was used to assess cell viability. Briefly, the medium was aspirated from the culture and MTT reagent was added to a final concentration of 0.5 mg/mL in culture medium. The samples were incubated for 3 h at 37 °C in the dark. Afterwards, the solution was aspirated, and the constructs were lysed using DMSO (D8418, Sigma-Aldrich, St. Louis, MO, USA). The absorbance was read at 550 nm using a microplate reader (Biotek ELX808, Winooski, VT, USA).

### 4.8. Real-Time Reverse Transcriptase-Polymerase Chain Reaction (RT-PCR)

RNA was extracted from the samples using peqGOLD total RNA kit (12-6834-02; PeqLab, Erlangen, Germany). After the removal of genomic DNA with Turbo DNA-free kit (AM1907; Invitrogen, Thermo Fisher Scientific, Waltham, MA, USA), cDNA was synthesized using High-Capacity cDNA Reverse Transcription Kit (4368814; Applied Biosystems, Thermo Fisher Scientific, Waltham, MA, USA). The cDNA obtained was analyzed by real-time reverse transcriptase-polymerase chain reaction (RT-PCR) using iQ SYBR Green Supermix (170-8884; Bio-Rad, Hercules, CA, USA) and primers designed for each gene of interest. The primers (Sigma-Aldrich, St. Louis, MO, USA) used were as follows: ribosomal protein L22 (RPL22,), forward 5′-TGACATCCGAGGTGCCTTTC-3′, reverse 5′-GTTAGCAACTACGCGCAACC-3′; collagen type I (COL1), forward 5′-AGACGGGAGTTTCTCCTCGG-3′, reverse 5′-CGGAGGTCCACAAAGCTGAA-3′; collagen type II (COL2), forward 5′-ATGACAATCTGGCTCCCAAC-3′, reverse 5′-CTTCAGGGCAGTGTACGTGA-3′; collagen type X (COL10), forward 5′-CCAATGCCGAGTCAAATGGC-3′, reverse 5′-GGGGGAAGGTTTGTTGGTCT-3′; aggrecan, (ACAN) forward 5′-TGGTGATGATCTGGCACGAG-3′, reverse 5′-CGTTTGTAGGTGGTGGCTGT-3′; SOX9, forward 5′-CAGACGCACATCTCCCCCAA-3′, reverse 5′-GCTTCAGGTCAGCCTTGCC-3′; RUNX2, forward 5′-GGTTCAACGATCTGAGATTTGTGGG-3′, reverse 5′-CACTGAGGCGGTCAGAGAACAAACTAG-3′ (all human). The real-time PCR was carried out under the following conditions: 10 min at 95 °C followed by 40 cycles of 15 s at 94 °C, 30 s at 55 °C (for COL2 and RUNX2) or 60 °C (for RPL22, COL1 and COL10) or 62 °C (for SOX9) or 64 °C (for ACAN), and 30 s at 72 °C. Finally, a melting step was performed from 58 °C to 95 °C to obtain the melting curve. Relative gene-fold variations were determined according to the 2^−∆∆Ct^ method using the RPL22 as a housekeeping gene. Ct values relative to ribosomal protein L22 (RPL22) were reported as fold increase (ΔΔCt) relative to 2D cultures at passage 6 in expansion medium (baseline).

### 4.9. Western Blot

Samples were lysed in RIPA buffer (R0278; Sigma-Aldrich, St. Louis, MO, USA), with protease inhibitor cocktail (Complete Mini, 11836153001; Roche, Basel, Switzerland). Acrylamide gels were prepared according to the size of the proteins, generally in concentrations of 7.5% or 10% (w/v). Cell lysates (5 mg of each sample) were run by applying 150 V during 90 min. After the run, proteins were transferred to a polyvinylidene difluoride (PVDF) membrane (LC 2005; Invitrogen, Thermo Fisher Scientific, Waltham, MA, USA) by applying 40 V over 2 h at room temperature. The membrane was incubated at room temperature for 2 h in blocking buffer (BB) consisting of 4% (w/v) nonfat powdered milk in PBST (phosphate buffer saline with Tween-20). Membranes were incubated for 1 h at room temperature with primary antibodies at a final concentration of 1 μg/mL in PBST. Then, a secondary antibody immunoglobulin G-horseradish peroxidase (IgG-HRP) was added, in a concentration of 1 μg/mL, and incubated at room temperature for 1 h. Finally the membrane was revealed for HRP detection with SuperSignal West Pico Chemiluminescent Substrate (34080; Thermo Fisher Scientific, Waltham, MA, USA). Chemiluminescent images were acquired in the ImageQuant™ LAS 4000 mini (GE HealthCare, Barcelona, Spain). Primary antibodies used were anti-Actin (1:400, sc-1615; SCBT, Dallas, TX, USA), anti-Collagen I (1:10,000, ab138492; Abcam, Cambridge, UK), anti-Collagen II (1:1000, ab3092; Abcam, Cambridge, UK) and anti-Collagen X (1:1000, ab182563; Abcam, Cambridge, UK). Secondary antibodies used were anti-goat IgG-HRP (1:1000, ab97100; Abcam, Cambridge, UK), anti-mouse IgG-HRP (1:1000, ab97023; Abcam, Cambridge, UK), and anti-rabbit IgG-HRP (1:1000, ab97051; Abcam, Cambridge, UK).

### 4.10. Toluidine Blue Staining

3D constructs were fixed with paraformaldehyde 2% (w/v) and incubated with toluidine blue (T3260, Sigma-Aldrich, St. Louis, MO, USA) 0.05% (w/w) in water during 20 min. Then, samples were washed several times with distilled water and, finally, visualized under a stereoscopic microscope (Leica M165 C, Barcelona, Spain).

### 4.11. Mechanical Characterization

A compression assay with DMA Multi-Frequency-Strain mode was applied to acellular PCL-based scaffolds and to each 3D construct cultured over 4 weeks with a DMA Q800 (TA Instruments, New Castle, DE, USA). The conditions of the assay were: Amplitude = 1 µm, Preload force = 0.01 N and Frequency = 1 Hz. The selected frequency is the standard working frequency used in this type of experiments and the selected amplitude is within a range of amplitude values where the sample remained constant. Construct diameter and thickness were measured for each sample. Results were obtained with TA Instrument Explorer software and analyzed with TA Universal analysis software. Values of storage modulus (G’), loss modulus (G’’), complex modulus (G*), and tan delta were obtained and represented in separated graphics. G’ is the measure of the sample’s elastic behavior, G’’ measures the viscous response of the material, G* is the sum of both components and, finally, the ratio of the loss to the storage is the tan delta and it is a measure of the energy dissipation of the material.

### 4.12. Statistics

All values were expressed as mean ± SD. Statistical differences were analyzed with GraphPad Prism 6 when samples were prepared in triplicate in each experiment for the condition analyzed. Statistical analysis was carried out by one-way or two-way ANOVA, as appropriate, followed by Tukey post analysis. N refers to independent experiments and n to samples per group in each experiment.

## 5. Conclusions

In this study, a new synthetic composite scaffold was obtained by infiltrating a 3D woven PCL scaffold with the RAD16-I self-assembling peptide hydrogel with cells (AC). While the woven microfiber PCL scaffold provides mechanical support, the nanofibers of RAD16-I peptide facilitate cell attachment and growth, resulting in a multi-scale biomimetic scaffold for CTE applications. The *in vitro* 3D culture of dedifferentiated human articular chondrocytes demonstrated that the new composite supports cell survival and promotes the re-establishment of the chondrogenic lineage commitment. The microenvironment provided to the embedded chondrocytes aims to resemble the native ECM by accomplishing mechanical and biological requirements of cartilage tissue. Therefore, this composite scaffold may provide a 3D culture platform for future therapeutic applications for cartilage repair or regeneration.

## Abbreviations

The following abbreviations are used in this manuscript:

3D	Three-dimensional;
PCL	Poly (ε-caprolactone);
RAD	RAD16-I peptide;
ECM	Extracellular matrix;
CTE	Cartilage tissue engineering;
FDA	Food and Drug Administration;
PEG	Polyethylene glycol;
IPN	Interpenetrating network;
SEM	scanning electron microscope;
AC	Articular chondrocytes;
MTT	3-(4,5-dimethylthiazol-2-yl)-2,5-diphenyltetrazolium bromide;
TGF β1	transforming growth factor-β1;
AA2P	L-Ascorbic Acid 2-phosphate;
COL1	Collagen type I;
COL2	Collagen type II;
COL10	Collagen type X;
ACAN	Aggrecan;
RPL22	Ribosomal protein L22;
qRT-PCR	quantitative Reverse Transcription Polymerase Chain Reaction;
GAGs	glycosaminoglycans;
PFA	Paraformaldehyde;
DMA	Dynamic Mechanical Analysis;
G’	storage modulus;
G’’	loss modulus;
G*	complex modulus;
PBST	phosphate buffer saline with Tween-20
